# Mosmo Is Required for Zebrafish Craniofacial Formation

**DOI:** 10.3389/fcell.2021.767048

**Published:** 2021-10-22

**Authors:** Carlos Camacho-Macorra, Marcos Sintes, Noemí Tabanera, Irene Grasa, Paola Bovolenta, Marcos J. Cardozo

**Affiliations:** ^1^Centro de Biología Molecular Severo Ochoa, Consejo Superior de Investigaciones Científicas, Universidad Autónoma de Madrid, Madrid, Spain; ^2^Centro de Investigación Biomédica en Red de Enfermedades Raras, Instituto de Salud Carlos III, Madrid, Spain

**Keywords:** hedgehog signaling (Hh), Smoothened (Smo), tetraspan transmembrane protein, craniofacial abnormalities, Mosmo

## Abstract

Hedgehog (Hh) signaling is a highly regulated molecular pathway implicated in many developmental and homeostatic events. Mutations in genes encoding primary components or regulators of the pathway cause an array of congenital malformations or postnatal pathologies, the extent of which is not yet fully defined. Mosmo (Modulator of Smoothened) is a modulator of the Hh pathway, which encodes a membrane tetraspan protein. Studies in cell lines have shown that Mosmo promotes the internalization and degradation of the Hh signaling transducer Smoothened (Smo), thereby down-modulating pathway activation. Whether this modulation is essential for vertebrate embryonic development remains poorly explored. Here, we have addressed this question and show that in zebrafish embryos, the two *mosmo* paralogs, *mosmoa* and *mosmob*, are expressed in the head mesenchyme and along the entire ventral neural tube. At the cellular level, Mosmoa localizes at the plasma membrane, cytoplasmic vesicles and primary cilium in both zebrafish and chick embryos. CRISPR/Cas9 mediated inactivation of both *mosmoa* and *mosmob* in zebrafish causes frontonasal hypoplasia and craniofacial skeleton defects, which become evident in the adult fish. We thus suggest that *MOSMO* is a candidate to explain uncharacterized forms of human congenital craniofacial malformations, such as those present in the 16p12.1 chromosomal deletion syndrome encompassing the *MOSMO* locus.

## Introduction

Communication among cells is a fundamental mechanism for the development of multicellular organisms. This communication is mostly mediated by elaborated signaling mechanisms, among which the Hedgehog (Hh) pathway represents a prototypical example. This pathway is evolutionary conserved and pleiotropically used among species (Ingham et al., 2011). Indeed, its function has been involved in a wide variety of developmental events including cell specification, proliferation, differentiation, migration, and axon guidance as well as in adult tissues’ homeostasis and regeneration (Sánchez-Camacho and Bovolenta, 2009; Briscoe and Thérond, 2013; Petrova and Joyner, 2014). These functions are exerted in different tissues and organs: among others, the central nervous system (CNS), the limbs, the vascular system, and the craniofacial structures (Abramyan, 2019; Sasai et al., 2019).

Hedgehog signaling relies on the widespread participation of core components of the pathway such as the transmembrane proteins Patched (Ptc) and Smoothened (Smo). By default, Ptc blocks the function of Smo that remains localized in endosomes. Upon Hh ligand binding, Ptc releases Smo inhibition, enabling Smo localization at the primary cilium of the targeted cell, thereby initiating the activation of specific intracellular cascades (Murone et al., 1999). The diversification and specificity of the signaling outputs is instead fostered by the participation of other components that have more restricted spatio-temporal distributions and/or can modify intracellular signaling in a context dependent manner. These include, for example, the ligands themselves [i.e., Sonic (Shh), Indian (Ihh), and Desert (Dhh) hedgehog], a number of Hh binding proteins such as Boc, Cdon, and Gas1 that can act both as positive (Cole and Krauss, 2003; Allen et al., 2007, 2011) or negative signaling regulators (Bergeron et al., 2011; Cardozo et al., 2014; Echevarría-Andino and Allen, 2020) and transcriptional or non-transcriptional effectors of the pathway (e.g., Gli1, Gli2, Gli3, PKA, and Src) (Jia et al., 2004; Sánchez-Camacho and Bovolenta, 2009; Yam et al., 2009; Hui and Angers, 2011). This diversity also explains the broad spectrum of congenital malformations (e.g., holoprosencephaly, ciliopathies, skeletal, and craniofacial defects) associated with mutations in gene encoding components of the Hh pathway (Sasai et al., 2019) or its defective postnatal function, which has been associated with a large number of cancer types (Jeng et al., 2020).

Whether we have unveiled the full extent of the Hh pathway complexity and of the pathologies associated to its dysfunction is still undetermined. Indeed, a recent genome-wide screen aimed at identifying novel modulators of Hh signaling using CRISPR/Cas9 technology in the NIH-3T3 mouse cell line, uncovered the existence of new pathway regulators, including an unannotated gene, now known as *MOSMO* (MOdulator of SMOothened) (Pusapati et al., 2018). In the same study, *Mosmo* was demonstrated to encode a membrane tetraspan protein, which promoted the endocytosis of the Hh transducer Smo, thereby lowering its levels at the cell plasma membrane (Pusapati et al., 2018). To what extent Mosmo participates in Hh signaling regulation *in vivo*, however, it is just beginning to be elucidated (Lasser et al., 2020; Kong et al., 2021; Pizzo et al., 2021).

Here, we have addressed this question and report that in zebrafish the two *mosmo* paralogs (*mosmoa* and *mosmob*) have an overlapping distribution in embryonic ventral neural tube and then in the larva head mesenchyme. Consistent with the latter distribution genetic inactivation of both paralogs causes frontonasal hypoplasia and craniofacial skeleton defects, suggesting that *MOSMO* is a candidate to explain uncharacterized forms of these type of human congenital malformations.

## Methods

### Fish Lines and Husbandry

AB/Tübingen (AB/Tue) zebrafish were maintained at 28°C on 14/10 h light/dark cycle. Embryos were raised at 28°C, collected and maintained in E3 medium (5 mM NaCl, 0.17 mM KCl, 0.33 mM CaCl, 0.33 mM MgSO4, 10^–5^% Methylene Blue). All used procedures were approved by the ethical committees for animal experimentation of the Consejo Superior de Investigaciones Científicas (CSIC) and Comunidad Autónoma de Madrid.

### Chick Embryos Maintenance

Fertilized chick embryos (Santa Isabel Farm, Cordoba, Spain) were incubated at 38°C in a humidified incubator until the desired stage, determined according to Hamburger and Hamilton (1992).

### Whole Mount *in situ* Hybridization

Total mRNA from AB/Tue zebrafish embryos was extracted using RNeasy Mini kit (Qiagen) according to manufacturer instructions. cDNA was synthesized using Super Script kit (Roche) following manufacturer instructions. PCR products, obtained from cDNA amplification using specific primers (Supplementary Table 1), were cloned in PCSA plasmid (Agilent Technologies), as described by the manufacturer. Plasmid DNA preparations were obtained using Genopure Plasmid Midi kit (Roche) following kit instructions. Digoxigenin-UTP-labeled antisense probes for *in situ* hybridization (ISH) were synthesized and purified using Super Script kit (Roche) following the manufacturer instructions. ISH was performed by standard procedures and visualized with NBT/BCIP (dark blue).

### Cloning Procedures

The PCSA-mosmoa_p1 plasmid was used as a template to amplify by PCR *mosmoa* and further add an hemagglutinin tag (HA) and restriction sites with the following primers: Fw 5′-aatCTCGAGCCTGAGATGGATAAACTC-3′. Rv 5′-ttaGAA TTCTCAAGCGTAATCTGGAACATCGTATGGGTAGCCAGG AAGACACACTTC-3′. The PCR product was cloned in PCSA plasmid (Agilent Technologies) as described by the manufacturer. The mosmoa-HA fragment was then excised with restriction enzymes and cloned in the pCIG vector (Megason and McMahon, 2002) for chick embryo electroporation and in pCS2 for cell transfection and synthesis of mRNA to be injected in zebrafish embryos.

### Chick Embryo Electroporation

The pCIG Mosmoa-HA plasmid (1 μg/μl) was co-injected with a pCAG-2A-Arl13b-tRFP [Arl13b-tRFP construct generated by Schmitz et al. (2017)] (1 μg/μl) into the neural tube ventricle of HH10 chick embryos followed by *in ovo* electroporation as previously described (Cardozo et al., 2014).

### Cell Transfection, Tissue Processing, and Immunochemistry

Human embryonic kidney (HEK) cells were cultured on glass coverslips in DMEM supplemented with 10% fetal calf serum and glutamine (2 mM). The pCS2-*mosmoa*-HA construct was transfected using lipotransfectin (Solmeglas) following the manufacture instructions. Cells were fixed with 4% paraformaldehyde in 0.1 M phosphate buffer pH 7.2 (wt/vol) at 37°C and then washed in PBS containing 0.5% Triton-X-100. Chick embryos were fixed by immersion in 4% cold PFA overnight at 4°C, washed, incubated in a 15% sucrose-PBS solution (wt/vol), embedded and frozen in a 7.5% gelatine in 15% sucrose solution (wt/vol). Cryostat sections, whole embryos or cells samples were stained with Hoechst and α-HA antibody produced in rabbit (1:250. Sigma, H-6908) and Donkey anti-Rabbit Alexa Fluor 488 secondary antibody (Invitrogen, A-21206), following standard procedures.

### Zebrafish Mutant Generation

Single guide RNAs (sgRNAs) targeting coding regions of *mosmoa* and *mosmob* for CRISPR/Cas9 deletion were designed using the tools provided by CHOPCHOP online service^1^ searching for potential disruption of restriction enzyme sites (Labun et al., 2019). Oligos were designed as described in Varshney et al. (2016) and their sequence is reported in Supplementary Table 1. sgRNAs were transcribed and purified using Maxi Script T7 (NEB) following the manufacturer’s instructions. sgRNAs were microinjected together with Cas9 protein (300 ng/μL; EnGen^®^ Spy Cas9 NLS, New England Biolabs) in 1 to 2 cell stage AB/Tue using a Narishige microinjector. F0 embryos were let grown and outcrossed with wt AB/Tue fish. Genomic DNA from tail clips of F1 zebrafish embryos was amplified by PCR and digested to identify disruption of selected restriction sites. DNA from potential mutants were sequenced, those with a disrupted and truncated reading frame were selected to generate the fish lines.

### Genotyping

DNA from embryos or adult fish was amplified by PCR using the primers listed in Supplementary Table 1. PCR products were digested with selected enzymes at 37°C for 2 h to distinguish among wt, heterozygous, and mutant *mosmoa* and/or *mosmob* fish.

### Bone and Cartilage Staining

Cartilage staining of zebrafish larvae and adult fish bones was performed with Alcian Blue and Alizarin Red, as, respectively described in Schilling et al. (1996), Sakata-Haga et al. (2018).

### Imaging and Data Processing

Embryos were immersed in 75% glycerol and whole-body images were obtained using a Leica CTR5000 stereomicroscope connected to a Leica DFC500 digital camera operated by Leica software. The adult fish stained with the Alizarin Red chromogen, which also emits red fluorescence, were photographed under fluorescent light stimulation using a Leica CTR5000 stereomicroscope connected to a Leica DFC350 FX digital camera operated by Leica software. The drawings in Figure 4G were traced in Adobe Illustrator using representative photographs of wt and *mosmoa*^–/–^;*mosmob*^–/–^ adult mutants. LSM710 confocal laser scanning coupled to an AxioObserver inverted microscope (Zeiss) was used to obtain digital images of cryostat sections or cells samples. ImageJ (Fiji) software was used to process and analyze images.

### Statistical Analyses

The ImageJ (Fiji) software was employed to obtain quantifications reported in Figure 3. Adult fish were anesthetized with tricaine and photographed in lateral views and the distance from the eye to the tip of the preorbital region was measured and normalized to the eye size in each one of the analyzed genotypes. Data were analyzed using GraphPad Prism 7 statistic software. One-way ANOVA test was used owing to the parametric distribution of the data, followed by Tukey’s multiple comparisons test to determine differences among groups.

## Results

### *Mosmo* Paralogs Show a Largely Overlapping Distribution in the Developing Zebrafish

**FIGURE 1:**
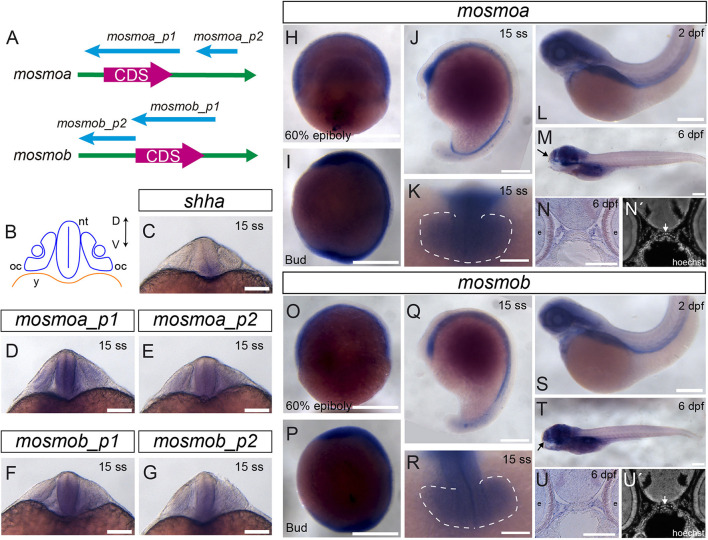
Mosmo paralogs show a largely overlapping distribution in zebrafish. **(A)** Schematic representation of *mosmoa* and *mosmob* mRNAs and probes used for *in situ* hybridization (ISH). p1, probe #1. p2, probe #2. **(B)** Schematic representation of a frontal section of a zebrafish embryo at 20 hpf at the level of the optic cup. Expression pattern of *shha* **(C)**, *mosmoa* **(D,E)** and *mosmob* **(F,G)** at 20 hpf. Note that the two probes for *mosmoa* and *mosmob* show an identical distribution. Note also that both paralogs have an overlapping distribution in the ventral neural tube but more dorsally extended than that of *shha*. **(H,U)** Expression pattern of embryos hybridized with *mosmoa* and *mosmob* at 60% epiboly **(H,O)**, bud **(I,P)** and 15 ss stage **(J,K,Q,R)**, 2 dpf **(L,S)** as well as at 6 dpf **(M,T)**. Note that at 60% epiboly and bud stage the expression of both genes is localized along the ventral anterior-posterior axis of the embryos. At 15 ss, a low level expression of both *mosmoa* and *mosmob* is detected in the optic vesicles (**K,R,** dashed line) and along the ventral neural tube from the diencephalon to the tail bud **(J,Q)**. At 2 and 6 dpf, the expression of both genes localizes to the head region. The eyes were removed in panels **(M,T)**. Frontal sections of 6 dpf embryos hybridized *in toto* for *mosmoa* **(N)** or *mosmob* **(U)** and counterstained with Hoechst **(N′,U′)**. ISH signal for both paralogs localizes around the ethmoid cartilage **(N,N′,U,U′** white arrows). D, dorsal; V, ventral; oc, optic cup; nt, neural tube; and y, yolk. Scale bars: 200 μM.

The zebrafish genome carries two different paralogs of the *mosmo* gene: *mosmoa* and *mosmob* (ZFIN:ZDB-GENE-101203-6; ZFIN:ZDB-GENE-060929-1030). To determine their expression pattern during embryonic and larval development, we generated two different specific ISH probes for each one of the two paralogs (Figure 1A). Both *mosmoa* and *mosmob* were detected at gastrulation and bud stages as well as during somitogenesis (Figures 1H,I,O,P). At this stage *shha*, one of the ligands of the pathway, is expressed along the midline of the entire ventral neural tube (Figures 1B,C), from which it diffuses to pattern the adjacent cells with a mechanism highly conserved across vertebrates (Martí et al., 1995; Roelink et al., 1995). During somitogenesis, *mosmoa* and *mosmob* were also found localized along the length of the ventral neural tube (Figures 1J,Q) with an overlapping distribution that, however, was more dorsally extended than that of *shha* (Figures 1C–G). Specific expression was also observed in the mesenchyme surrounding the neural tube (Figures 1D–G) and in the optic vesicles (Figures 1K,R). At 2 dpf and larval stages, *mosmoa* and *mosmob* were no longer detected in the neural tube but strongly localized in the head mesenchyme (Figures 1L,M,S,T), surrounding, among others, the ethmoid plate (Figures 1N,N′,U,U′).

The reported patterns were consistently observed with both of the probes generated for each one of the paralogs (Figure 1), validating the reported distribution.

### *M*osmoa Localizes at the Plasma Membrane, Endosomes, and Primary Cilia

**FIGURE 2:**
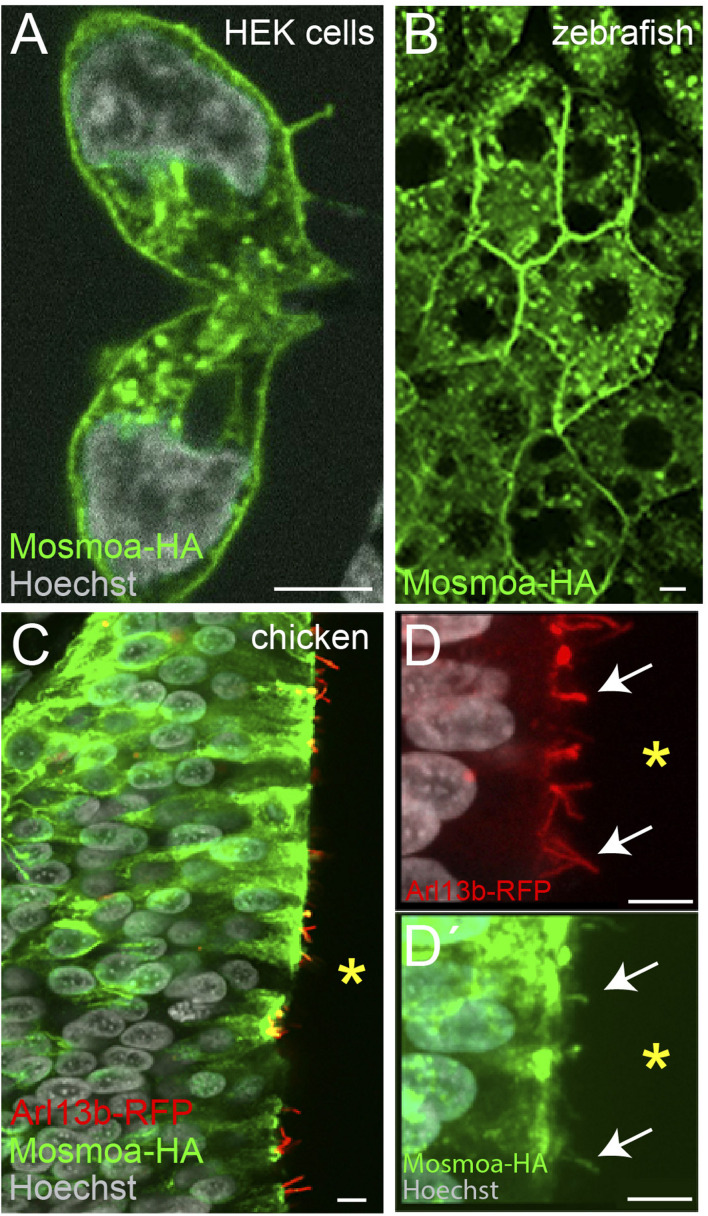
*M*osmoa localizes at the plasma membrane, endosomes, and primary cilia. **(A)** Example of human embryonic kidney (HEK) cells transfected with *mosmoa-HA*, immunostained for α-HA (green), and counterstained with Hoechst (white). **(B)** Dorsal view of a zebrafish gastrula (7 hpf) injected with *mosmoa-HA* mRNA and immunostained for α-HA (green). **(C–D′)** Transversal sections of chick embryo neural tubes co-electroporated with *mosmoa-HA* and the cilia marker *arl13b-RFP*. In both HEK cells and zebrafish EVL cells Mosmoa-HA signal localizes at the plasma membrane and in endo-vesicles. In HH14 chick embryos, neural tube Mosmoa-HA is also observed in the in the *Arl13b*-positive cilia (white arrows) **(D,D′)**. Yellow asterisk marks the neural tube ventricle **(C–D′)**. Scale bars: 5 μm.

Attempts to determine the subcellular localization of the protein showed that, at least in NIH-3T3 cells, Mosmo localizes at the plasma membrane and endosome (Pusapati et al., 2018). To verify if this is the subcellular distribution in the developing embryo, we generated a human influenza hemagglutinin (HA) tagged version of *mosmoa* (*mosmoa-HA*). We focused on this paralog because its amino acid (aa) sequence is 100% identical to that of its human ortholog, whereas mosmob aa sequence has a lower homology (89.8% identity). We first verified the efficiency of our construct by transfecting HEK cells with the *mosmoa-HA* containing plasmid followed by immunostaining for HA. As reported for NIH-3T3 cells (Pusapati et al., 2018), the tagged protein was detected at the plasma membrane and endosomes (Figure 2A). When *mosmoa-HA* mRNA was injected in zebrafish, HA immunosignal was similarly localized at the blastomers’ plasma membrane and endosomes (Figure 2B).

**FIGURE 3:**
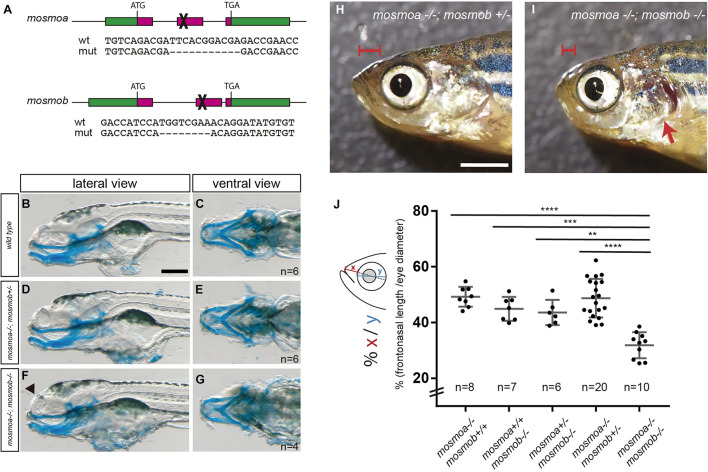
*mosmoa*^–^*^/^*^–^*;mosmob*^–^*^/^*^–^ double mutants display facial abnormalities. **(A)** Schematic representation of the strategy used to inactivate *mosmoa* and *mosmob* zebrafish genes using CRISPR-Cas9 technology and sequence of the selected mutants. **(B–G)** Lateral and ventral views of 5 dpf wild type **(B,C)**, *mosmoa*^–^*^/^*^–^*;mosmob^±^* **(D,E)** and *mosmoa*^–^*^/^*^–^*;mosmob*^–^*^/^*^–^ **(F,G)** zebrafish larvae stained with Alcian Blue to detect cartilage head organization. The rostral tip of the head appeared flatter in some of the double mutants (**F**, arrowhead). Eyes were removed for better staining visualization. The number of animals analyzed for each genotype is indicated in the right bottom corner in panels **(C,E,G)**. Scale bar 150 μm. **(H,I)** Lateral view of adult *mosmoa*^–^*^/^*^–^*;mosmob^±^* **(H)** and *mosmoa*^–^*^/^*^–^*;mosmob*^–^*^/^*^–^ double mutants **(I)**. Note that in double mutants the head is flatter and shorter (**I**, red brackets) than in *mosmoa*^–^*^/^*^–^*;mosmob^±^* fish and the operculum is abnormal exposing the gills (**I**, red arrow). **(J)** Quantification of the distance from the eye to the tip of the preorbital region (x) in relation to the eye size (y) in adult fish of different genotypes. *mosmoa*^–^*^/^*^–^*;mosmob*^–^*^/^*^–^ double mutants show a shorter fronto-nasal length than their siblings. One-way ANOVA followed by Tukey’s multiple comparison tests to analyze differences among groups. **P < 0.01, ***P < 0.001, and ****P < 0.0001. Scale bar 20 mm.

**FIGURE 4:**
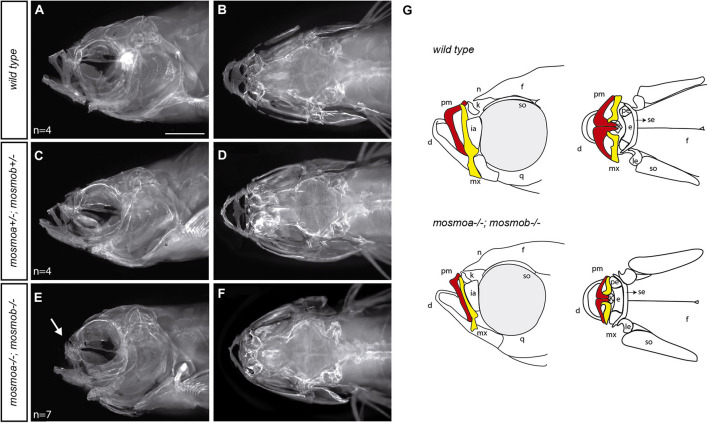
Mosmo paralogs are required for head bone formation in zebrafish. **(A–F)** Lateral **(A,C,E)** and dorsal **(B,D,F)** views of the head of wt **(A,B)**, *mosmoa^±^;mosmob^±^* **(C,D)** and *mosmoa*^–^*^/^*^–^*;mosmob*^–^*^/^*^–^ **(E,F)** adult zebrafish stained with alizarin red to label bone tissue. Note the bone malformation in the frontonasal region of *mosmoa*^–^*^/^*^–^*;mosmob*^–^*^/^*^–^ double mutants (**E**, arrow) in comparison to heterozygous and wt fish **(A,C)**. **(G)** Cartoons of the craniofacial appearance of wt and *mosmoa*^–^*^/^*^–^*;mosmob*^–^*^/^*^–^ adult mutants, from lateral and dorsal views, highlighting the maxillary (yellow) and premaxillary (red) bones. The number of animals analyzed for each genotype is indicated in the left bottom corner in panels **(A,C,E)**. d, dentary; e, ethmoid; f, frontal; ia, infraorbital; k, kinethmoid; le, lateral ethmoid; mx, maxillary; n, nasal; pe, pre-ethmoid; pm, premaxillary; q, quadrate; se, supraethmoid; and so, supraorbital. Scale bar, 2 mm.

When Hh signaling is active, Smo localizes at the primary cilium of the targeted cells. Notably, Mosmo was also observed in the primary cilia of NIH-3T3 cells (Pusapati et al., 2018). We thus asked if this localization could be observed also *in vivo*. The primary cilium can be easily detected in the chick neural tube as this organelle protrudes in the rather wide ventricle of chicken embryos. We thus co-electroporated two plasmids carrying *mosmoa-HA* and *arl13b-RFP*, respectively. The latter is a primary cilium specific protein, widely used to visualize this structure (Schmitz et al., 2017). Indeed, 24 h after electroporation, at HH14, HA, and RFP fluorescent signals co-localized in the cilium of a subset of the electroporated cells (Figures 2C–D′).

Taken together these data indicate that *in vivo* Mosmoa localizes at the plasma membrane, endosomes, and the primary cilia, suggesting that it may favors Smo translocation to this organelle, thus influencing signaling activation.

### Mosmo Paralogs Are Required for Zebrafish Craniofacial Formation

To explore the possible roles of *mosmoa* and *mosmob*, we inactivated the two genes using CRISPR-Cas9 technology. We selected founders at the F1 generation that carried frameshift mutations in either *mosmoa* or *mosmob* gene and generated stable *mosmoa***^–^**^/^**^–^** and *mosmob***^–^**^/^**^–^** mutant lines (Figure 3A). *Mosmoa***^–^**^/^**^–^** and *mosmob***^–^**^/^**^–^** mutant embryos show no gross morphological defects and grew to adulthood without evident defects. This was perhaps not surprising given that both genes share expression pattern and their respective proteins present a high degree of sequence homology, suggesting that the two paralogs may compensate each other activity. To overcome this possible compensatory effect, we intercrossed the *mosmoa***^–^***^/^***^–^** and *mosmob***^–^***^/^***^–^** mutant lines obtaining a *mosmoa***^–^***^/^***^–^***;mosmob***^–^***^/^***^–^** double mutant fish. At first glance, double mutant embryos showed no major gross alterations or histological defects along the neural tube and their size was similar to that of their sibling (not shown).

Other than in the neural tube, the two *mosmo* paralogs are expressed with a largely overlapping pattern also in different regions of the larva head. We thus used Alcian blue staining to label the cranio-facial cartilage of the larva. There were no obvious differences in the cartilaginous elements when wt, *mosmoa***^–^**^/^**^–^**;*mosmob***^–^**^/+^ and *mosmoa***^–^**^/^**^–^**;*mosmob***^–^**^/^**^–^** were compared (Figures 3B–G), although the rostral tip of the head appeared flatter at least in part of the double mutants (Figure 3F, arrowhead). To determine if this abnormality was only transient, we analyzed the morphology of the head in the adult fish. *Mosmoa***^–^**^/^**^–^**;*mosmob***^–^**^/^**^–^** double mutants consistently exhibited a significantly shorter frontonasal region (Figures 3H,I), which was not observed in their sibling of other genotypes (Figure 3J). Furthermore, the operculum was reduced in size, leaving the gills exposed (Figure 3I).

Hedgehog signaling is essential for the development of the anterior neurocranium (Wada et al., 2005) and disruption of *smo* activity in zebrafish affects the craniofacial skeleton (Eberhart et al., 2006; Swartz et al., 2012). Thus, the frontonasal hypoplasia observed in the double mutants could be the consequence of alterations in the osseous components of the craniofacial skeleton. To determine this possibility, we stained the skeleton of wt, *mosmoa*^±^;*mosmob*^±^ and *mosmoa***^–^**^/^**^–^**;*mosmob***^–^**^/^**^–^** adult fish with Alizarin red (Figures 4A–F). The bones of the frontonasal region, especially the maxillary and premaxillary bones of *mosmoa***^–^**^/^**^–^**;*mosmob***^–^**^/^**^–^** double mutants were altered as compared to those of wt or heterozygous fish (Figures 4A–F) as highlighted in the schematic drawings reporting the phenotypes (Figure 4G).

Taken together these data indicate that *mosmoa* and *mosmob* have an overlapping function, which is required for the acquisition of a proper craniofacial structure in zebrafish.

## Discussion

Modulators of Hh signaling play crucial roles in diversifying the output of Hh signaling (Gallardo and Bovolenta, 2018). The present study reinforces this idea and shows that in zebrafish the combined activity of the two *mosmo* paralogs, *mosmoa* and *mosmob*, are required for the proper craniofacial formation in zebrafish.

This apparently restricted effect is somewhat surprising as both *mosmoa* and *mosmob* are expressed with an overlapping pattern not only in the craniofacial mesenchyme of the larvae but also along the ventral region of the embryonic neural tube. The latter distribution overlaps with that of a number of Hh signaling components, including the ligand *shha*, *shhb* (Ekker et al., 1995), or the receptor *ptch2* (Concordet et al., 1996), and the transducer *smo* (Varga et al., 2001). In line with the idea that Mosmo acts on Smo promoting its endocytosis (Pusapati et al., 2018), we found Mosmoa localized in endocytic vesicles and the plasma membrane as well as the primary cilium, where Smo translocate when Hh signaling is activated. Thus and as previously proposed (Pusapati et al., 2018), the combined activity of the two *mosmo* paralogs could modulate Hh signaling activation in different contexts during development. However, loss of *mosmo* function in zebrafish seems to be mostly linked to the formation of the cranio-facial skeleton, with an evident head hypoplasia in the adult mutant fish but no other obvious defects. Indeed, *Mosmo* double mutants grow to adulthood and do not seem to have obvious behavioral problems, supporting a non-essential role of *mosmo* paralogs for zebrafish growth, survival, and reproduction. Consistent with this idea, we have not observed neural tube defects or gross abnormalities in other organs of the mutants at least upon histological analysis. Nevertheless, we cannot rule out the possibility that subtle defects may be found with a more in-depth analysis. Indeed, a recent study shows that, in mouse, *Mosmo* contributes to embryonic development and its loss of function causes skeletal, heart, and lung anomalies leading to embryonic lethality (Kong et al., 2021). However, coinciding with our observations, no defects in neural tube patterning were, however, found (Kong et al., 2021). Knock-down of *mosmo* in *Xenopus* instead shows a craniofacial phenotype in which both craniofacial and cartilage development appears affected, in association with alteration of neural crest cell proliferation and migration (Lasser et al., 2020). Interestingly, the coexistence of neurodevelopmental and craniofacial defects were observed in experiments performed in both *Drosophila* and *Xenopus* aimed at testing the importance of “a two-hit model” as trigger of neurodevelopmental disorders (Pizzo et al., 2021). Notably the study demonstrated a synergistic interaction between mutated *mosmo* and *setd5* (Pizzo et al., 2021), a gene encoding a histone methyltransferase, which has been associated with intellectual disability (Grozeva et al., 2014). This functional interaction observed in both *Drosophila* and *Xenopus* seems to be present also in humans (Pizzo et al., 2021). In this respect the *mosmoa***^–^**^/^**^–^**;*mosmob***^–^**^/^**^–^** double mutants, could be an additional model in which to explore how *setd5* and *mosmo* synergize causing more severe congenital malformations.

The Hh ligands Shh and Ihh are osteogenic regulators and both are expressed in craniofacial elements (Chiang et al., 1996; Pan et al., 2013). In both mouse and zebrafish, Ihh secreted by chondrocytes stimulates the ossification of the perichondrial cell layer that surrounds the developing cartilage (St-Jacques et al., 1999; Hammond and Schulte-Merker, 2009). In mice, conditional inactivation of *ihh* in cranial neural crest cells causes skeletal malformations, including a markedly hypoplastic nasomaxillary complex (Amano et al., 2020). Furthermore, zebrafish mutants lacking enzymes involved in proteoglycans synthesis (*fam20b***^–^***^/^***^–^** and *xylt1***^–^***^/^***^–^**) exhibit an accelerated *ihh* expression and premature bone formation, resulting in an adult fish with midface hypoplasia among other malformations (Eames et al., 2011). These features resemble those we observe in the *mosmoa***^–^***^/^***^–^***; mosmob***^–^***^/^***^–^** double mutants. Thus, it is tempting to speculate that *mosmo* paralogs may participate in signaling response triggered by *ihh* during osteogenesis, perhaps with an accelerated bone formation in the absence of Mosmo activity.

Intraflagellar proteins (IFTs) in the primary cilia, such as IFT80, affect Hh signaling and are required for osteoblast differentiation (Yuan et al., 2016). The craniofacial/skeletal abnormalities linked to Mosmo function in *Xenopus* (Lasser et al., 2020; Pizzo et al., 2021), mouse (Kong et al., 2021), and zebrafish (this study) together with MOSMO protein subcellular localization, suggest that *MOSMO* homologs could be key controllers of SMO translocation to the primary cilia during osteogenesis, thereby modulating signal transduction. Although worthwhile testing, this possibility remains at the moment a speculation, given the lack appropriate genetic tools that enable following protein movements within the cilium.

Independently of the precise pathway components with which Mosmo may function, the coincidence of some phenotypic features observed upon inactivation of *Mosmo* in *Xenopus* (Lasser et al., 2020; Pizzo et al., 2021), mouse (Kong et al., 2021), and zebrafish (this study) suggests that defective function of the human *MOSMO* may have similar consequences. Notably, a deletion in chromosome 16, encompassing the human *MOSMO* among others genes, causes a rare disease known as recurrent 16p12.1 deletion syndrome. Patients present developmental delay, intellectual disability, and other anomalies which may vary from individual to individual. Among these anomalies, craniofacial and skeletal are among the most frequently found defects (Girirajan et al., 2010), including microcephaly and flat face (Ballif et al., 2007; Girirajan et al., 2010), resembling, at some point, the phenotype observed in adult *mosmoa***^–^***^/^***^–^***;mosmob***^–^***^/^***^–^** mutants.

## Conclusion

In conclusion, our study shows a restricted and overlapping distribution of *mosmo* genes in zebrafish revealing the subcellular localization of the Mosmoa protein during development in endosomes, plasma membrane, and primary cilia. More importantly, the generation of *mosmoa***^–^***^/^***^–^***;mosmob***^–^***^/^***^–^** zebrafish mutants provides support for the idea that the human *MOSMO* might be a candidate gene underlying uncharacterized forms of rare congenital craniofacial malformations. The double mutants further provide the opportunity to dissect the contribution of *MOSMO* to the phenotype associated with the human 16p12.1 deletion syndrome.

## Data Availability Statement

The original contributions presented in the study are included in the article/Supplementary Material, further inquiries can be directed to the corresponding authors.

## Ethics Statement

The animal study was reviewed and approved by the Consejo Superior de Investigaciones Científicas (CSIC) and Comunidad Autónoma de Madrid.

## Author Contributions

MC and PB conceptualized and designed the research study and wrote the manuscript. MS and CC-M performed the ISH analysis and mutant generation and characterization. NT and MC generated data reported in Figures 2, 3 and IG in Figure 4. PB obtained the financial support. All authors read and approved the manuscript.

## Conflict of Interest

The authors declare that the research was conducted in the absence of any commercial or financial relationships that could be construed as a potential conflict of interest.

## Publisher’s Note

All claims expressed in this article are solely those of the authors and do not necessarily represent those of their affiliated organizations, or those of the publisher, the editors and the reviewers. Any product that may be evaluated in this article, or claim that may be made by its manufacturer, is not guaranteed or endorsed by the publisher.

## Footnotes

1
http://chopchop.cbu.uib.no
