# Supplementary material for: Mosmo Is Required for Zebrafish Craniofacial Formation
Source: Front Cell Dev Biol. 2021 Oct 22;9:767048. doi: 10.3389/fcell.2021.767048 (PMC8569894; doi:10.3389/fcell.2021.767048)
Supplement: Supplementary file 1 [file Table_1.DOCX]

**Mosmo is required for zebrafish craniofacial formation**

Carlos Camacho-Macorra^1,2, †^, Marcos Sintes^1,^ ^†^, Noemí Tabanera^1,2^, Irene Grasa^1^, Paola Bovolenta^1,2,*^ and Marcos J. Cardozo^1,2,*^

^1^Centro de Biología Molecular Severo Ochoa, Consejo Superior de Investigaciones Científicas-Universidad Autónoma de Madrid, Madrid 28049, Spain. ^2^Centro de Investigación Biomédica en Red de Enfermedades Raras (CIBERER), Instituto de Salud Carlos III (ISCIII), Madrid 28049, Spain

**Supplementary Table 1.** List of primers and sgRNAs used in this study

| **mRNA probes** | **Primer Fw** | **Primer Rv** | | **Size (bp)** |
| --- | --- | --- | --- | --- |
| *mosmoa_p1* | CCAAACACACCGAGAGCATC | TGCAGCGATCGTCTTTTCAG | | 923 |
| *mosmoa_p2* | GAGTTTTAGGGAGCACTGCG | GCAATGTCGTCCACCACTTT | | 694 |
| *mosmob_p1* | CTGACTGAAGTGTGAAAATGG | ATAACTCCCTTTGGATGACAC | | 772 |
| *mosmob_p2* | CGAGCAGGGTTGTGTTTTGA | CCATTTTCACACTTCAGTCAGT | | 523 |
|  | | | | |
| **sgRNAs** |  | |  | |
| *mosmoa*: | ´-taatacgactcactataGGGCAGTGTCAGACGATTCAgttttagagctagaaatagc-3´ | |  | |
| *mosmob*: | 5´-taatacgactcactataGATCCTGTTTCGACCATGGAgttttagagctagaaatagc-3´ | |  | |
| Universal_bottom-strand-ultramer | 5´AAAAGCACCGACTCGGTGCCACTTTTTCAAGTTGATAACGGACTAGCCTTA  TTTTAACTTGCTATTTCTAGCTCTAAAAC-3´ | |  | |
|  | | | | |
| **Genotyping** | **Primer Fw** | **Primer Rv** | | **Enzyme** |
| *mosmoa* | TGCTGCATGTAATTGATTGTTG | AGGTGACTGTGAGGGAGATGAT | | Hinf I |
| *mosmob* | TTCATGTGTTTTGCATCTAGGG | TGATCAACCTACTTCCCATGAA | | NcoI |
